# Nematode surface functionalization with hydrogel sheaths tailored *in situ*

**DOI:** 10.1016/j.mtbio.2022.100328

**Published:** 2022-06-16

**Authors:** Wildan Mubarok, Masaki Nakahata, Masaru Kojima, Shinji Sakai

**Affiliations:** Division of Chemical Engineering, Department of Materials Engineering Science, Graduate School of Engineering Science, Osaka University, Osaka, 560-8531, Japan

**Keywords:** Surface functionalization, *Caenorhabditis elegans*, *Anisakis simplex*, Hydrogel sheath, Living drug delivery system, Horseradish peroxidase

## Abstract

Engineering the surfaces of biological organisms allows the introduction of novel functions and enhances their native functions. However, studies on surface engineering remained limited to unicellular organisms. Herein, nematode surfaces are engineered through *in situ* hydrogelation mediated by horseradish peroxidase (HRP) anchored to nematode cuticles. With this method, hydrogel sheaths of approximately 10-μm thickness are fabricated from a variety of polysaccharides, proteins, and synthetic polymers. *Caenorhabditis elegans* and *Anisakis simplex* coated with a hydrogel sheath showed a negligible decrease in viability, chemotaxis and locomotion. Hydrogel sheaths containing UV-absorbable groups and catalase functioned as shields to protect nematodes from UV and hydrogen peroxide, respectively. The results also showed that hydrogel sheaths containing glucose oxidase have the potential to be used as living drug delivery systems for cancer therapy. The nematode functionalization method developed in this study has the potential to impact a wide range of fields from agriculture to medicine.

## Highlights

• Technique to modify the surface of nematode with hydrogel is presented.
• Hydrogel is fabricated *in situ* using enzymatic cross-linking reaction.
• The technique is compatible with different materials and different species of nematodes.
• Hydrogel sheath protects the nematode from environmental stress.
• Nematode coated with drug-loaded hydrogel has a potential as living drug delivery system.

## Graphical abstract

**Figure:**
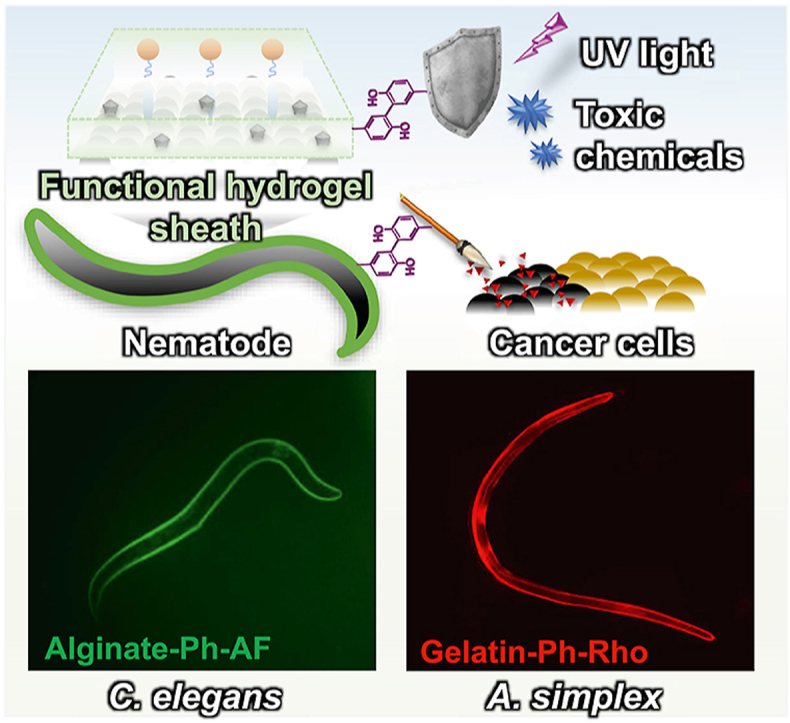

## 1 Introduction

The surface of biological organisms is important as a barrier against environmental stress, as well as a contact point between signal and receptor proteins that govern the behavior, and even survival, of organisms. Recent advances in encapsulation technology have allowed the modification of cell surfaces with various materials, for instance*,* mammalian cells with hydrogels [1], yeast with iron nano-shells [2], and *Chlorella* and *Daphnia magna* with titanium oxide shells [3,4]. These surface modifications have allowed the introduction of new functions and have also enhanced the original functions of the cell. Previous studies have utilized cell-surface engineering for targeted isolation and migration [5,6], protection against oxidants, environmental stress, lysing agents [7,8], surface imaging [9], target recognition [10,11], and drug-loading [12,13].

Although numerous studies have reported the surface modification of unicellular organisms, surface modification of multicellular organisms is rarely conducted. Multicellular organisms, such as nematodes, are more closely related to humans and have a complex anatomy and physiological function. They also live in more diverse environments compared to unicellular organisms. Functionalizing nematodes, therefore, could have an impact in biomedical, environmental, and industrial fields. In light of this, a surface modification technique was developed to functionalize the nematode by loading materials onto its surface.

To the best of our knowledge, only one previous study has developed a technique to modify the surface of nematode: Minullina et al. (2014) modified the surface of *Caenorhabditis elegans* (*C. elegans*) with polyelectrolyte shells obtained through a layer-by-layer deposition of polycations and polyanions [14]. However, this technique was limited by the necessity to use cationic polymers. Cationic polymers often exhibit toxicity owing to their interactions with the cell membranes [15]. Moreover, direct contact between the negatively charged membrane and positively charged molecules may also turn on random endocytosis pathways for these materials to cross the membrane [16].

**Fig. 1:**
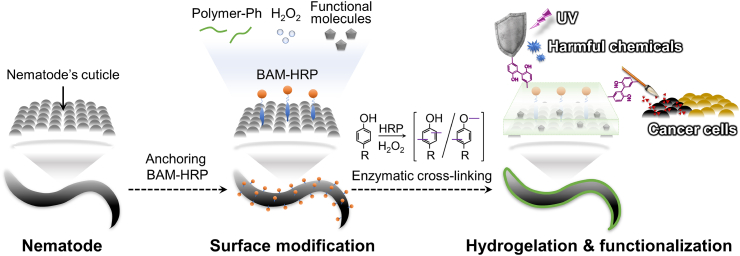
Framework of *in situ* cross-linking technique on nematode's cuticle (CTICLE). *In situ* enzymatic cross-linking is achieved by anchoring horseradish peroxidase (HRP) to the surface of nematode via biocompatible anchor membrane (BAM-HRP) that allows hydrogel sheath fabrication of polymer possessing phenol groups (Polymer-Ph) in the presence of hydrogen peroxide (H_2_O_2_). Hydrogel sheath modification allows the functionalization of the nematode to kill cancer cells and shield the nematode against environmental stress.

In this study, we demonstrated the surface functionalization of nematode with various hydrogels with different properties for the first time. We developed an *in situ* cross-linking technique on a nematode cuticle (CTICLE) to functionalize the surface of nematodes by fabricating a hydrogel sheath from biocompatible polymers on their surface (Fig. 1). We focused on two different species of nematodes, *C. elegans* and *Anisakis simplex* (*A. simplex*). *C. elegans* is a soil worm and was the first multicellular organism to have its whole genome sequenced, with 65% of its genes having homologs with genes associated with human diseases [17,18]. These homologous genes make *C. elegans* a suitable model for biomedical research models including toxicity screening, drug development, and cancer drug screening. Moreover, *A. simplex* has been reported to have the potential ability to sense cancer chemically and attach to cancerous tissues [[19], [20], [21], [22]]. This opens up a possibility to introduce a variety of new functions to the nematode by modifying its surface with hydrogel sheath.

To fabricate a stable on-cuticle hydrogel sheath, horseradish peroxidase (HRP) was conjugated with an oleyl chain derivative coupled with polyethylene glycol, known as a biocompatible anchor for membrane (BAM). HRP conjugated with BAM (BAM-HRP) allows the immobilization of HRP to the cuticle of the nematode. HRP-mediated cross-linking of polymers possessing phenol groups (Polymer-Ph) in the presence of H_2_O_2_ is a well-established method that has been used for various purposes such as cell encapsulation, 3D bioprinting, and *in situ* wound healing [[23], [24], [25]]. Sakai et al. reported the use of BAM-HRP to modify mammalian cells with hydrogel sheaths with 1-μm thickness [1,23,26]. Zhao et al. (2020) recently reported a similar system to sheath RhD-negative red blood cells to achieve universal blood transfusion [27]. To demonstrate the feasibility of CTICLE, we coated the surfaces of *C. elegans* and *A. simplex* with hydrogel sheaths composed of Ph-modified alginate (Alginate–Ph), gelatin (Gelatin–Ph), and poly(vinyl alcohol) (PVA-Ph). We evaluated the effect of the coating on the viability, chemotaxis, and locomotion of these nematodes. Functionalization of the nematode surfaces was examined by evaluating the functions of individual hydrogel sheaths as a protective shield against UV light and H_2_O_2_ and as a spear allowing the nematode to be a potential living cancer drug.

## 2 Methods

### 2.1 Materials

Tyramine hydrochloride was purchased from Chem-Impex International (Wood Dale, IL, USA). 2-Morpholinoethanesulfonic acid (MES) was purchased from Dojindo Molecular Technologies (Kumamoto, Japan). Horseradish peroxidase (HRP, 190 U ​mg^−1^), hydrogen peroxide (H_2_O_2_) aqueous solution (31% w/w), *N*-hydroxysuccinimide (NHS), *N,N*-dimethyl-formamide (DMF), catalase from bovine liver, and pepsin (1:60000, porcine stomach mucosa), and d-glucose were purchased from Wako Pure Chemical Industries (Osaka, Japan). Glucose oxidase was purchased from BBI Enzymes (Torfaen, UK). BAM-NHS (Sunbright, OE-080CS) was purchased from NOF (Tokyo, Japan). Water-soluble carbodiimide hydrochloride (WSCD·HCl) was purchased from the Peptide Institute (Osaka, Japan). Sodium alginate (Kimica I-1G, MW 70 ​kDa, high guluronic acid content) was purchased from Kimica (Tokyo, Japan). Gelatin (bovine skin, ca. 225 g bloom, type B) and NHS-rhodamine were purchased from Sigma-Aldrich (St. Louis, MO, USA). Lithium phenyl(2,4,6-trimethylbenzoyl)phosphinate (LAP), 3-(4-hydroxyphenyl) propionic acid, 1,5-EDANS hydrate, and 5-aminofluorescein were purchased from Tokyo Chemical Industries (Tokyo, Japan). PVA-COOH (AF-17, 19.6% vinyl repeat units) was kindly gifted by Japan Vam & Poval Co., Ltd. (Osaka, Japan). Titanium (IV) sulfate solution (Ti(SO_4_)_2_, 5%) was purchased from Nacalai Tesque (Kyoto, Japan). Dulbecco's modified Eagle's medium (DMEM) and RPMI 1640 were purchased from Nissui (Tokyo, Japan).

### 2.2 Preparation of Polymer-Ph

Alg-Ph-Aminofluorescein (Alg-Ph-AF: 1.5 ​× ​10^−4^ ​mol-Ph g^−1^) and 1,5-EDANS-labeled PVA-Ph (PVA-Ph-EDANS: 4.3 ​× ​10^−5^ ​mol-Ph g^−1^) were prepared by conjugating tyramine hydrochloride to alginate with 5-aminofluorescein and PVA-COOH with 1,5-EDANS, respectively, via NHS/WSCD·HCl chemistry [25,28]. Gelatin-Ph-Rhodamine (Gela-Ph-Rho: 2.4 ​× ​10^−4^ ​mol-Ph g^−1^) and Catalase-Ph (1.7 ​× ​10^−4^ ​mol-Ph g^−1^) were prepared by conjugating 3-(4-hydroxyphenyl)propionic acid with NHS and WSCD·HCl, as reported previously [29]. The amount of Ph group was observed based on the peak at 275 ​nm plotted against the tyramine hydrochloride standard (Supplementary Fig. S1).

### 2.3 Preparation of BAM-HRP

BAM-HRP was prepared using the methods reported in previous studies [1,30]. Briefly, a phosphate-buffered saline (PBS) solution containing 0.3% w/v HRP was mixed with 1.2% w/v BAM-NHS dissolved in dimethylsulfoxide at a 95:5 ​vol ratio. After mixing for 2 ​h at room temperature (24–27 ​°C), BAM-HRP was collected by centrifugation at 14,000×*g* for 30 ​min at 4 ​°C using a molecular weight cutoff of 10 ​kDa. The BAM-HRP content was determined using Bradford protein assay (Supplementary Fig. S2). The obtained BAM-HRP was then stored at 4 ​°C in PBS solution.

### 2.4 *C. elegans* culture

The *C. elegans* wild-type N2 strain was propagated on nematode growth media (NGM; 1.7% w/v agar, 0.25% w/v peptone, 50 ​mM NaCl, 5 ​mg ​mL^−1^ cholesterol, 1 ​mM CaCl_2_, 1 ​mM MgSO_4_, 25 ​mM KPO_4_) plates seeded with *Escherichia coli* OP50 as a food source [31,32].

### 2.5 *A. simplex* culture

*A. simplex* was collected from chub mackerel (*Scomber japonicus*), blue mackerel (*Scomber australasicus*), and Pacific saury (*Cololabis saira*) caught in the Sea of Japan. *A. simplex* larvae stage 3 (L3) were isolated from the digestive organs of the host fish (Supplementary Fig. S3). The nematodes were then rinsed several times with phosphate-buffered saline (PBS). Each larva was individually placed in an antibiotic-antimycotic solution for 30 ​min. *A. simplex* was then cultured in RPMI-1640 medium containing 20% v/v heat-inactivated fetal bovine serum, 0.5 ​mg ​mL^−1^ pepsin at pH 5.6 in a 37 ​°C humidified incubator supplied with 5% CO_2_ [[33], [34], [35]].

### 2.6 Fabrication of hydrogel sheath

Nematodes were isolated from the culture medium and washed twice with PBS. The nematodes were then immersed in BAM-HRP solution for 10 ​min. After washing twice with PBS, the nematodes were immersed in a solution containing H_2_O_2_ and fluorescently labeled Polymer-Ph for 10 ​min. The nematodes were then washed twice with PBS and observed using a fluorescence microscope (BZ-9000, Keyence, Tokyo, Japan) and a confocal laser scanning microscope (C2, Nikon, Tokyo, Japan). The thickness and fluorescence intensity of the hydrogel sheath were measured using ImageJ software (1.46r; NIH, Bethesda, MD, USA).

### 2.7 Chemotaxis assay

Chemotaxis analysis was conducted by assessing the chemotactic response of *C. elegans* toward isoamyl alcohol (IAA). A chemotaxis assay was conducted on a 10-cm assay plate (2.0% w/v agar, 1 ​mM CaCl_2_, 1 ​mM MgSO_4_, 25 ​mM KPO_4_ buffer, thickness 4 ​mm) according to previous studies [36,37]. On one end of the plate (point A), 2 ​μL of 1.0 v/v% IAA in ethanol mixed with 1 M NaN_3_ (1:1) was placed, while on the opposite end (point B), the same volume of ethanol mixed with 1 ​M NaN_3_ (1:1) was added. *C. elegans* was then placed at the center of the assay plate. The number of *C. elegans* on each side was counted after 60 ​min. *C. elegans* that remained within 1 ​cm of the starting point were excluded from the study. The chemotaxis index was calculated as (*N*_A_ ​− ​*N*_B_)/(*N*_A_ ​+ ​*N*_B_).

### 2.8 Locomotion analysis

Locomotion analysis was conducted by measuring the average speed of *C. elegans*. *C. elegans* locomotion on the assay plate was recorded at 60 fps using a camera mounted on a stereo microscope. Locomotion analysis was conducted using the wrMTrck plugin (Build 110622) in the ImageJ software [38].

### 2.9 Nematode viability post-coating

The viability of *C. elegans* and *A. simplex* was investigated by monitoring the viability of the nematodes before and after BAM-HRP coating and post-hydrogel sheath coating. The viability of nematodes was determined based on the movement response following mechanical or touch stimuli [[39], [40], [41]].

### 2.10 UV protection

UV–Vis light absorbance of sodium alginate, 0.1% w/v Alg-Ph-AF solution, and 1.0% w/v Alg-Ph-AF hydrogels were measured using UV–Vis spectroscopy (UV-2600, Shimadzu, Kyoto, Japan) in the wavelength range of 220–600 ​nm. *C. elegans*, coated with 1.0% w/v Alg-Ph-AF hydrogel sheath or non-coated as a control, were transferred to assay plates and then exposed to UV-C (254 ​nm) with varying degrees of energy: 0, 250, 500, and 1000 ​J ​m^−2^. The viability of *C. elegans* was directly observed following UV exposure based on its movement response after mechanical stimulation.

### 2.11 Protection against hydrogen peroxide

*C. elegans* was coated with Alg-Ph-AF hydrogel containing Catalase-Ph, obtained by sequentially incubating *C. elegans* in 12 ​μg ​mL^−1^ BAM-HRP followed by immersion in a solution containing 1.0% w/v Alg-Ph-AF, 1.0% w/v Catalase-Ph, and 0.1 ​mM ​H_2_O_2_. *C. elegans* was then immersed in 200 ​μL of 1 M H_2_O_2_ solution in a 96-well plate for 60 ​min. Non-coated *C. elegans* and *C. elegans* coated with hydrogel without Catalase-Ph were used as controls. The test was conducted in triplicate with at least 15 worms per test. *C. elegans* viability was assessed at 0, 1, 10, 30, and 60 ​min based on movement after mechanical stimuli.

### 2.12 NAGOX treatment on cancer cells

HeLa cells were cultured in a 6 well-plate at 1.0 ​× ​10^3^ ​cells cm^−2^. After overnight culture, gelatin methacrylate (GelMA) hydrogel was fabricated on top of the cells by adding 1 mL PBS solution containing 5.0% w/v GelMA and 0.5% w/v LAP followed by irradiation with 405-nm blue light for 40 ​s. Cells were then cultured in culture medium (control, DMEM containing 10% v/v FBS) or co-cultured with one *A. simplex* coated with 1.0% w/v Alg-Ph-AF and loaded with 100 ​μg ​mL^−1^ GOX (NAGOX) in culture medium for 24 ​h. Apoptotic cells were observed after staining with 3.3 ​μg ​mL^−1^ propidium iodide (PI) in PBS for 10 ​min using a fluorescence microscope. The number of apoptotic cells was counted using the ImageJ software. H_2_O_2_ production by GOX in the hydrogel sheath of NAGOX was measured by Ti(SO_4_)_2_-based colorimetry. Briefly, a NAGOX was put in 3 ​mL PBS containing 1 ​mg ​mL^−1^ d-glucose. Periodically, 100 ​μL aliquot of the solution was collected and H_2_O_2_ content in the solution was measured based on the colorimetric determination using Ti(SO_4_)_2_ [42,43].

### 2.13 Statistical analysis

Data were tabulated and analyzed using Microsoft® Excel® 2019 (version 1808; Microsoft Corp., Redmond, WA, USA). Data were analyzed using one-way analysis of variance (ANOVA). Post hoc *t*-tests were conducted using Tukey's HSD. Data were considered statistically significant when *p* ​< ​0.05.

## 3 Results and discussion

### 3.1 Hydrogel sheath fabrication

To determine the effects of *in situ* hydrogel sheath formation on nematode surfaces, the composition of nematode immersion solutions and the order of immersion in the solutions were investigated in *C. elegans*. The hydrogel sheath was observed by fluorescence microscopy using the fluorescent-labeled Polymer-Ph on the surface of the nematode.

**Fig. 2:**
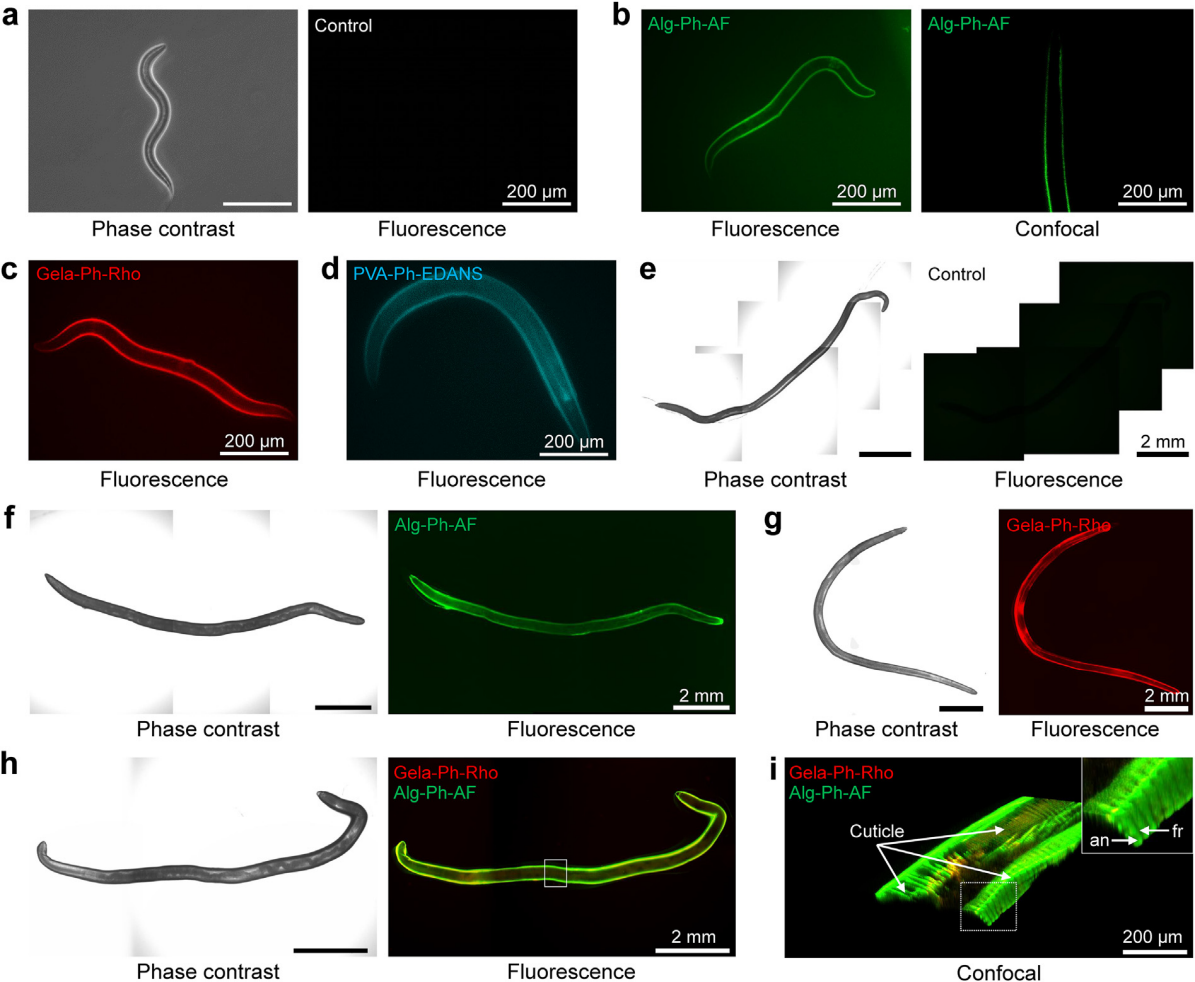
Engineering the surface of (a–d) *Caenorhabditis elegans* and (e–i) *Anisakis simplex* obtained through *on-cuticle* enzymatic cross-linking. (a, e) Control test in which nematode was immersed in Alg-Ph-AF ​+ ​H_2_O_2_ without prior immersion in BAM-HRP. Nematode coated with hydrogel sheath fabricated by sequential immersion in BAM-HRP and H_2_O_2_ ​+ ​(b, f) Alg-Ph-AF or (c, g) Gela-Ph-Rho. (d) *C. elegans* coated with PVA-Ph-EDANS. (h) Fluorescence and (i) confocal observation of *A. simplex* coated with double-layered hydrogel consisted of Gela-Ph-Rho and Alg-Ph-AF. Insert showing annulus (an) and furrow (fr).

First, the possibility of fabricating a hydrogel sheath on the surface of *C. elegans* using native HRP was investigated to determine the necessity of conjugation of BAM with HRP for immobilizing the enzyme on the surface of nematodes. The *C. elegans* immersed in a solution containing 5–190 U mL^−1^ HRP, followed by immersion in a solution containing 1.0% w/v Alginate-Ph labeled with aminofluorescein (Alg-Ph-AF) and 0.1 ​mM ​H_2_O_2_, showed no fluorescence attributed to Alg-Ph-AF (Supplementary Fig. S4). Alg-Ph-AF hydrogel sheath was also not fabricated on the surface of *C. elegans* immersed in a solution containing Alg-Ph-AF and H_2_O_2_ alone (Fig. 2a). In contrast, the *C. elegans* immersed sequentially in a solution containing 12 ​μg ​mL^−1^ BAM-HRP and the solution containing 1.0% w/v Alg-Ph-AF and 0.1 ​mM ​H_2_O_2_ showed the fluorescence attributed to Alg-Ph-AF hydrogel sheath (Fig. 2b). Confocal laser-scanning microscope observation showed that the hydrogel sheath was localized on the nematode surface (Fig. 2b; Supplementary Fig. S5). The thickness of Alg-Ph-AF hydrogel sheath was 8.0 ​± ​0.4 ​μm (means ​± ​S.E.) (Supplementary Fig. S6). This hydrogel is thicker than the reported value of 250 ​nm to 1 ​μm for the cell coating fabricated using HRP-mediated cross-linking [1,27]. This difference could be attributed to the higher surface density of BAM-HRP molecules introduced to the surface of nematodes compared to that of cells. Additionally, the nematode cuticle differs from the cell surface, in which the nematode cuticle consists of numerous ring-like structures called the annulus [44,45]. The cell membrane has an even surface with a thickness of 3 ​nm [46], whereas the uneven surface of the nematode has depth differences of approximately 40–100 ​nm [47]. This uneven surface leads to BAM-HRP anchoring at different angles and positions, which, in turn, results in the formation of a thicker hydrogel compared to the cell membrane (Supplementary Fig. S7).

Furthermore, the results were not specific to Alg-Ph-AF, as hydrogel sheaths were also obtained from Gelatin-Ph labeled with rhodamine (Gela-Ph-Rho) (Fig. 2c) and poly(vinyl alcohol)-Ph labeled with 5-(2-aminoethylamino)-1-naphthalenesulfonic acid (PVA-Ph-EDANS) (Fig. 2d). These results demonstrate the necessity of immobilizing HRP on the surface of nematodes by using BAM-HRP before immersion in a solution containing Polymer-Ph and H_2_O_2_, and the possibility of preparing hydrogel sheaths with different functions from a variety of materials cross-linkable through HRP-mediated reactions.

To further demonstrate the versatility of this technique, a hydrogel sheath was fabricated on the surface of another nematode species, *A. simplex*. *A. simplex* is a marine nematode and parasite that uses various fish as a host and has been known to accidentally use humans as hosts. A previous study has reported that the cuticle of *A. simplex* consists of lipids, with fatty acids being the most abundant fraction [48]. This lipid layer allows the anchoring of BAM-HRP, which, in turn, allows hydrogel sheath fabrication on the surface of the nematode*.*

Similar to the results obtained for *C. elegans*, no hydrogel sheath was formed on the *A. simplex* immersed in a solution containing Polymer-Ph and H_2_O_2_ alone (Fig. 2e) but formed through sequential immersion in a solution containing BAM-HRP and a solution containing Alg-Ph-AF or Gela-Ph-Rho and H_2_O_2_ (Fig. 2f and g). Additionally, a double-layered dual-material hydrogel sheath was fabricated by sequentially immersing the nematodes in solutions containing Alg-Ph-AF and Gela-Ph-Rho (Fig. 2h). Confocal laser-scanning microscopy observation of the *A. simplex* coated with Alg-Ph-AF and Gela-Ph-Rho (Fig. 2i) hydrogel sheaths revealed the detailed structure of the cuticle, including the annulus and furrow, demonstrating the possibility of using the double-layered hydrogel sheath coating for on-demand cuticle observation.

### 3.2 Effects of hydrogel sheath coating on the behavior of nematodes

**Fig. 3:**
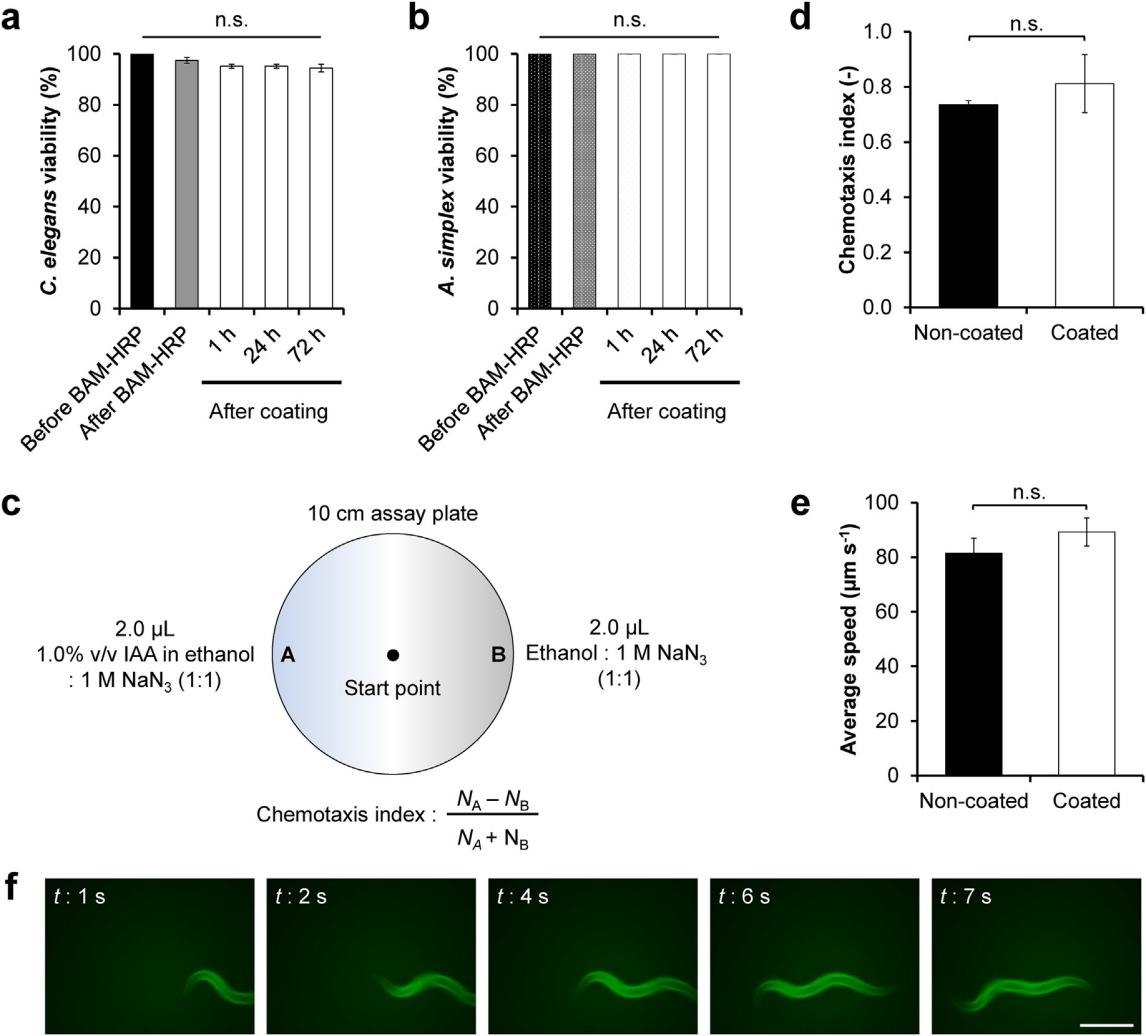
Effect of on-cuticle cross-linking of Alg-Ph-AF hydrogel on viability and physiological functions of the nematodes. (a) Viability of *C. elegans* (*n* ​= ​5 tests) and (b) *A. simplex* (*n* ​= ​3 tests) before (black column) and after (grey column) BAM-HRP immobilization, and 1–72 ​h after hydrogel sheath formation (white column). (c–d) Effect of the hydrogel sheath on chemotaxis. (c) Experimental setup for chemotaxis assay. (d) Chemoattraction of *C. elegans* non-coated or coated with the hydrogel sheath to 1.0% v/v isoamyl alcohol (IAA) in ethanol (*n* ​= ​3 tests). (e) Comparison of the average locomotion speed of non-coated and coated *C. elegans* (*n* ​≥ ​27 worms). Bar: S.E. n.s.: *p* ​> ​0.05. (f) Fluorescence observation of the locomotion of *C. elegans* coated with Alg-Ph-AF hydrogel. Scale bar: 200 ​μm.

Next, the effects of *in situ* hydrogel sheath formation through HRP-mediated hydrogelation on the viability and physiological functions, such as chemotaxis and locomotion of nematodes, were investigated. While the H_2_O_2_ used in this hydrogelation method might intuitively possess a toxic effect, its concentration used in this study (0.1 ​mM) showed the minimum toxic effect on *C. elegans* with >90% viability after 10 ​min of incubation and 80% viability after 60 ​min (Supplementary Fig. S8). *C. elegans* also showed high viability (>90%) before and after BAM-HRP coating as well as 1–72 ​h after coating (Fig. 3a). A similar trend was also observed in *A. simplex* (Fig. 3b), indicating that the CTICLE technique had a negligible effect on nematode viability.

We then investigated the effect of the hydrogel sheath coating on the chemotactic behavior of nematodes. Wild-type *C. elegans* is highly attracted to 1.0% v/v isoamyl alcohol (IAA) in ethanol and shows movement in response to stimulus [49,50]. The *C. elegans* coated with the Alg-Ph-AF hydrogel sheath showed an attracted response to IAA. No significant difference in the chemotaxis index (defined as shown in Fig. 3c) was found between the values detected in *C. elegans* with and without the hydrogel sheath (Fig. 3d, *p* ​> ​0.05). This result indicates that the diffusion of low-molecular-weight compounds, such as IAA, is not prevented in the hydrogel network, which allows the detection of IAA by the sensory neurons of *C. elegans*. Molecular diffusion through the hydrogel has also been reported in an Alg-Ph hydrogel obtained by HRP-mediated cross-linking, showing low-molecular-weight compounds freely diffuse in the hydrogels almost similar as in aqueous solutions [51]. This might explain how *C. elegans* still has the ability to ‘smell’ the chemicals in its surrounding environment, despite the hydrogel sheath coating.

Furthermore, the hydrogel sheath did not slow down the locomotion speed compared to that of non-coated *C. elegans* (Fig. 3e; Supplementary Mov. S1). Fluorescence observation of the hydrogel-coated *C. elegans* showed that the hydrogel sheath remained on the cuticle surface despite locomotion on the agar plate (Fig. 3f; Supplementary Mov. S2). Further observation showed that the hydrogel sheath remained on the surface of *C. elegans* for 3-days after coating (Supplementary Fig. S9). It is likely that the stable presence of the hydrogel sheath on the surface of the nematode is caused by the thickness of the hydrogel. Thin hydrogel films are generally known to have higher flexibility than thick hydrogels, which are easily broken down by bending (Supplementary Fig. S10) [52]. Taken together, these results indicate that the CTICLE technique is harmless to nematodes with minimal effects on viability and physiological functions. This finding is in accordance with previous studies that reported the biocompatibility of the HRP-mediated cell encapsulation technique using both homogenously dissolved HRP [29] and anchored HRP [1].

Supplementary data related to this article can be found online athttps://doi.org/10.1016/j.mtbio.2022.100328.

The following are the Supplementary data related to this article:

Multimedia component 1 Multimedia component 1

Multimedia component 2 Multimedia component 2

### 3.3 Functional hydrogel sheaths

As mentioned above, the CTICLE technique allows the development of hydrogel sheaths from various materials on nematode surfaces with minimal effects on viability, chemotaxis, and locomotion. This means that the CTICLE technique has the potential to make nematodes even more useful by providing functions to the hydrogel sheath by incorporating functional molecules such as enzymes and chemicals suitable for each application.

**Fig. 4:**
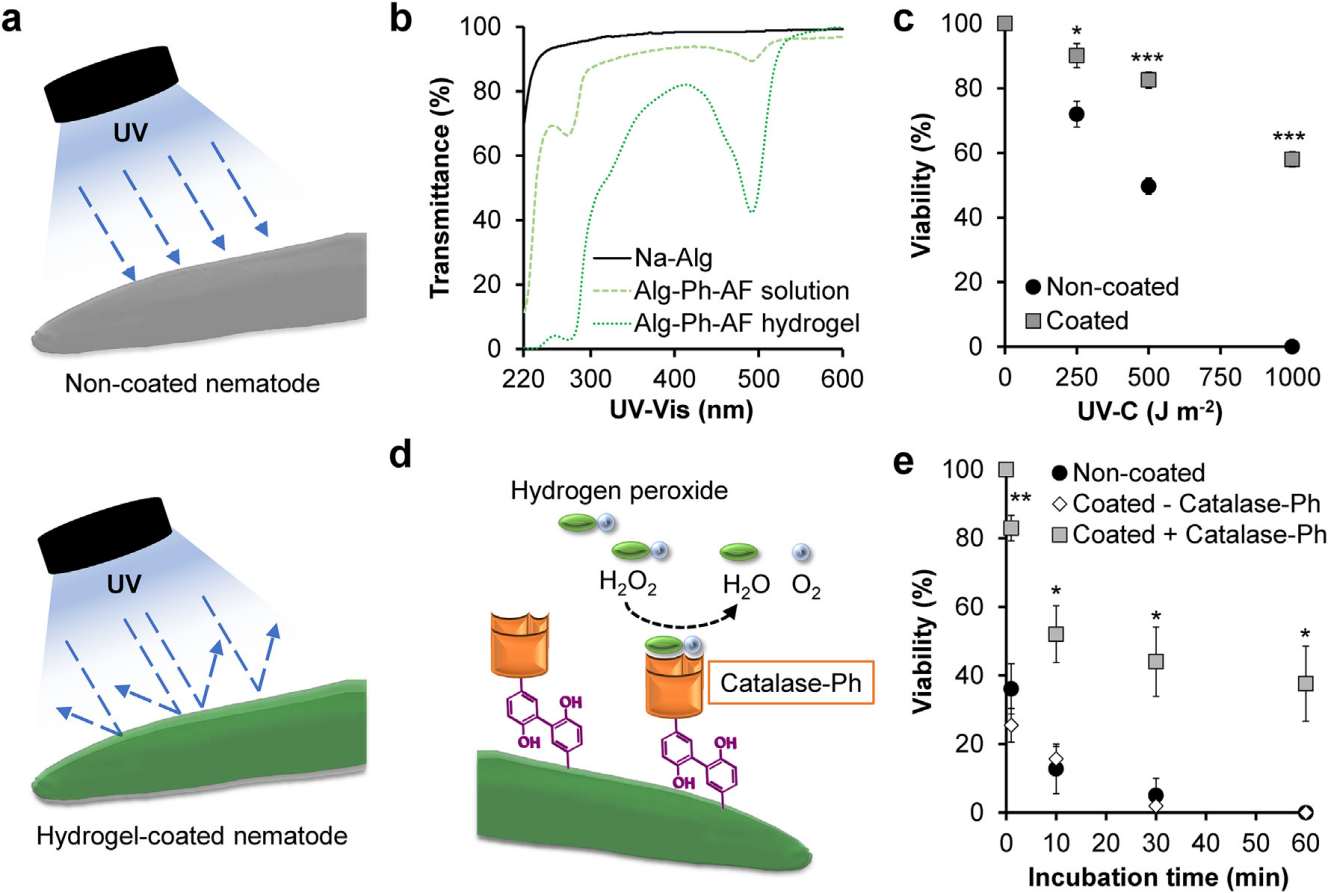
Protective function of hydrogel sheath against environmental stress. (a) Shielding function of hydrogel sheath against UV-C. (b) UV and visible light (UV–Vis, 220–600 ​nm) transmittance of sodium alginate (Na-Alg), Alg-Ph-AF solution and hydrogel. (c) Viability of *C. elegans* coated or non-coated with Alg-Ph-AF hydrogel sheath exposed to UV-C (λ: 254 ​nm, *E*: 0–1000 ​J ​m^−2^). Bar: S.E. (*n* ​= ​6 tests) (d) Schematic illustration of *C. elegans* protection against hydrogen peroxide (H_2_O_2_) by incorporating Catalase-Ph to the hydrogel sheath. (e) Viability of *C. elegans* coated with Alg-Ph-AF hydrogel sheath loaded with Catalase-Ph following immersion in 1 ​M ​H_2_O_2_ for 60 ​min. *C. elegans* non-coated or coated with hydrogel sheath without Catalase-Ph was used as control. Bar: S.E. (*n* ​= ​3 tests). ∗*p* ​< ​0.05, ∗∗*p* ​< ​0.005, ∗∗∗*p* ​< ​0.0005, compared to non-coated, Tukey HSD.

As a proof-of-concept, we studied hydrogel sheaths that protect nematodes in their sheaths from UV exposure and hydrogen peroxide. The hydrogel sheath prevents the transmission of UV light (Fig. 4a). In comparison with the transmission of UV light through sodium alginate solution, the transmission prevention by Alg-Ph-AF hydrogel can be explained by the existence of non-cross-linked or cross-linked Ph groups (Fig. 4b). The ability of the Ph groups to absorb UV light is well known [28,29]. Viability analysis showed dose-dependent toxicity in non-coated *C. elegans* exposed to 1000 ​J ​m^−2^ of UV-C (254 ​nm; 0–1000 ​J ​m^−2^) (Fig. 4c). In contrast, *C. elegans* coated with the Alg-Ph-AF hydrogel sheath had a higher viability of up to 60% at the highest dose of 1000 ​J ​m^−2^. These results demonstrated the Alg-Ph-AF hydrogel functions as a shield against harmful UV light in *C. elegans*.

In addition to the protective function of UV light, further functionalization can provide the hydrogel with additional defensive capabilities. A substantial amount of H_2_O_2_ was introduced into the soil by both wet and dry atmospheric deposition [53]. H_2_O_2_ is widely used in the removal of organic matter in soil remediation [54,55], and H_2_O_2_ treatment is harmful to biological organisms living in the soil, including *C. elegans* [56]. Catalase is known to exert a protective effect against hydrogen peroxide by degrading it into oxygen and water [51,57].

Therefore, we incorporated a catalase derivative containing phenol moieties (Catalase-Ph) into the Alg-Ph-AF hydrogel sheath via HRP-mediated conjugation of these two molecules (Fig. 4d). Conjugation of two different molecules possessing Ph moieties has also been reported [58]. The introduction of Ph moieties to catalase did not induce changes in its activity (Supplementary Fig. S11). As expected, the resultant hydrogel sheath functioned as a shield to protect *C. elegans* from H_2_O_2_, showing higher viability than that of non-coated *C. elegans* (Fig. 4e). Bubble formation was also observed specifically in H_2_O_2_-containing *C. elegans* coated with a hydrogel sheath containing Catalase-Ph (Supplementary Fig. S12), indicating degradation of H_2_O_2_ to oxygen and water in the surrounding area of *C. elegans*. Based on these results, a hydrogel sheath containing functional molecules could have a shielding effect against various environmental stresses.

**Fig. 5:**
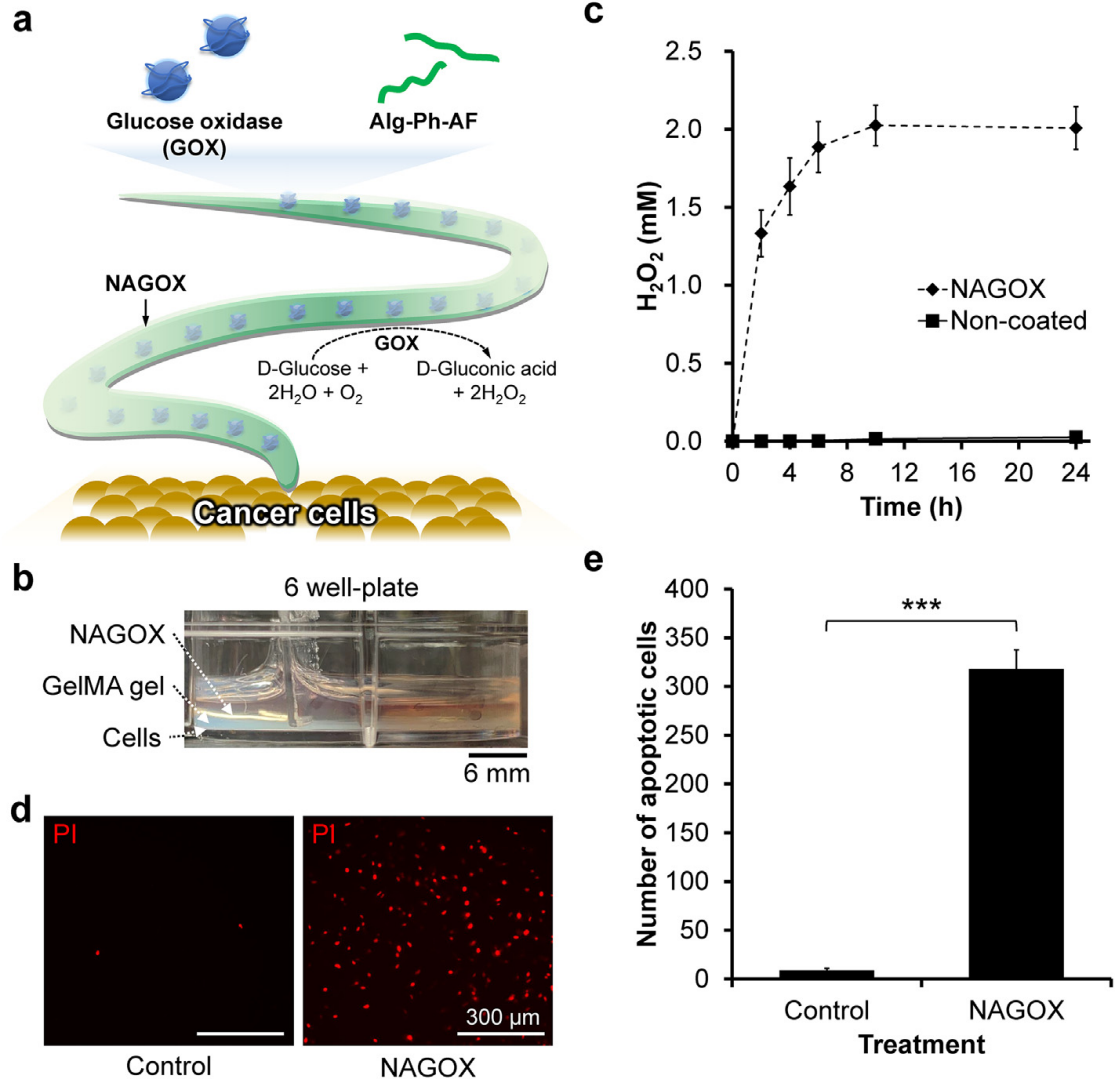
Functionalization of nematode as living drug delivery system for cancer treatment. (a) Schematic illustration of nematode coated with hydrogel sheath loaded with glucose oxidase (NAGOX) to induce cancer cells killing. (b) *In vitro* experiment setup in 6 well-plate. (c) H_2_O_2_ release by NAGOX compared with non-coated *A. simplex*. Bar: S.E. (*n* ​= ​3). (d) Fluorescence observation of apoptotic HeLa cells stained with propidium iodide (PI). (e) Number of apoptotic HeLa cells cultured in normal culture medium (control) and co-cultured with NAGOX. Bar: S.E. (*n* ​= ​6). ∗∗∗*p* ​< ​0.0005, Tukey HSD.

Among the various candidate molecules that can be incorporated into the hydrogel sheath to make it function as a spear, in this study, we attempted to incorporate glucose oxidase (GOX). Previous studies have immobilized GOX in nanogels [59], vesicles [60], and nanoparticles [61] for cancer treatment. Although there are several issues that must be overcome, such as allergic reactions against parasite antigens and on-demand removal after the achievement of the objective, as a proof-of-concept, we studied the potential ability of the nematode with the sheath as a living drug delivery system for cancer treatment (Fig. 5a).

Multiple clinical studies have reported the presence of *Anisakis* in human gastrointestinal cancer [[19], [20], [21]] with a recent study suggesting that *Anisakis* may sense the cancer chemically and attach to the cancerous tissue due to changes in mucin around the cancer [22]. GOX catalyzes the conversion of d-glucose into d-gluconic acid and hydrogen peroxide (H_2_O_2_) (Supplementary Fig. S13). Depletion of glucose as a cancer cell energy source can cause cancer cells to die, whereas the generated H_2_O_2_ can kill cancer cells as an oxidant [60,62,63]. Oxidation of glucose by GOX also affects the tumor environment in the form of enhanced hypoxia due to the consumption of O_2_ and increased acidity by the production of d-gluconic acid [64].

We studied the efficiency of the hydrogel sheath containing GOX in killing HeLa cells after checking the dose-dependent induction of their death by GOX through apoptosis (Supplementary Fig. S14). To eliminate cell detachment caused by direct contact with the moving *A. simplex*, HeLa cells adhering to cell culture dishes were layered with a methacrylated gelatin (GelMA) hydrogel of 2-mm thickness fabricated through conventional photo-cross-linking using LAP (Fig. 5b; Supplementary Fig. S15). Before the study using HeLa cells, we investigated the H_2_O_2_ production profile by GOX contained in the hydrogel sheath by putting NAGOX in a solution containing 1 ​mg ​mL^−1^ d-glucose. The content of H_2_O_2_ in the solution increased with extending the incubation time during the first 10 ​h and then reached a constant value, 2 ​mM, in the presence of NAGOX (Fig. 5c). In contrast, H_2_O_2_ did not increase in the presence of non-coated *A. simplex*. The result showing constant value after producing a certain amount of H_2_O_2_ was not specific to the GOX contained in the hydrogel sheath. Similar result was found for free GOX dissolved in the solution containing 1 ​mg ​mL^−1^ d-glucose (Supplementary Fig. S16). It can be explained by the inactivation of GOX by H_2_O_2_ accumulated in the solution [[65], [66], [67]]. The H_2_O_2_ concentration produced by NAGOX is higher than those reported to induce cell death or damage in HeLa, lung cancer and gastric adenocarcinoma cell line [[68], [69], [70]]. Yet, we found no remarkable changes in the movement of NAGOX after 24 ​h of the study. Fluorescence observation of propidium iodide (PI)-stained cells (Fig. 5d) showed that HeLa cells co-cultured with NAGOX for 24 ​h showed 36 times more PI-stained cells compared to control cells (Fig. 5e). These results indicated that NAGOX induced apoptosis in HeLa cells. Taken together, nematode coating with a GOX-loaded hydrogel sheath could be a promising method for developing a living drug delivery system for cancer treatment with high efficiency.

In general, we have successfully demonstrated the functionalization of the nematode surface with hydrogel sheath that could act as both the shield and the spear against environmental stresses and possible cancer drug delivery implications. While this is a promising result, future studies are required to address several issues. In this study, the enzyme immobilized in the sheath of the hydrogel maintained its activity. However, the introduction of the Ph moiety may decrease the activity of some enzymes. Additionally, chemical introduction of Ph groups to enzymes implies that the Ph group is introduced randomly. An alternative approach to avoid these issues may be to introduce tyrosine in areas where genetic modification does not induce a loss of activity. To realize the clinical application of functionalized *A. simplex* in the future, it would be necessary to develop strains that do not produce allergens, similar to hypoallergenic wheat and eggs [71,72]. Gastro-allergic reactions caused by *A. simplex* is well known [73,74]. It is also necessary to develop anthelmintic for *A. simplex* to eradicate it from patients at the appropriate time. Development of this anthelminthic also could be beneficial to treat anisakiasis caused by the accidental ingestion of *A. simplex*. Moreover, if the size of the nematode changes with growth, the membrane may break. Therefore, it is suggested to use nematodes at a stage in which the developmental stage has already stabilized with minimal changes in the size afterwards. Further applications of the hydrogel sheath to other nematode species also needs to be addressed in the future. For instance, the root-knot nematode *Meloidogyne incognita* and *Rotylenchulus reniformis* that could be used to deliver drugs or bacteria to plant roots could have applications in agriculture.

## 4 Conclusion

The method of nematode coating with hydrogel sheaths presented in this study has great potential. A hydrogel sheath is fabricated *in situ* on the surface of the nematode mediated by HRP anchored on the cuticle of the nematode, which catalyzes the cross-linking of Polymer-Ph in the presence of hydrogen peroxide. The versatility of this technique was demonstrated using a variety of polymers possessing Ph moieties. In addition, our technique can be applied to different species of nematodes originating from different habitats. The hydrogel sheath can shield nematodes against environmental stress. Moreover, hydrogel sheath coating allows the functionalization of nematodes as a potential living drug delivery system for cancer treatment. The method proposed in this study can extend the application of surface engineering technology and has a wide range of possible target organisms, including other important nematode species or lipid-coated multicellular organisms with industrial and biomedical applications.

## Credit author statement

W.M.: Formal analysis, Validation, Data curation, Visualization, Writing – original draft, Writing – Reviewing and Editing. M.N., M.K., S.S.: Conceptualization, Resources, Project administration, Methodology, Supervision, Writing – original draft, Writing – Reviewing and Editing.

## Data availability statement

The data that support the findings of this study are available from the corresponding author upon reasonable request.

## Declaration of competing interest

The authors declare that they have no known competing financial interests or personal relationships that could have appeared to influence the work reported in this paper.
