# Supplementary material for: Nematode surface functionalization with hydrogel sheaths tailored in situ
Source: Mater Today Bio. 2022 Jun 16;15:100328. doi: 10.1016/j.mtbio.2022.100328 (PMC9237936; doi:10.1016/j.mtbio.2022.100328)
Supplement: Supplementary file 3 [file mmc3.docx]

Supplementary Information

Nematode Surface Functionalization with Hydrogel Sheaths Tailored *In Situ*

Wildan Mubarok, Masaki Nakahata, Masaru Kojima, Shinji Sakai*

Division of Chemical Engineering, Department of Materials Engineering Science,

Graduate School of Engineering Science, Osaka University, Osaka, 560-8531, Japan

*Corresponding author: sakai@cheng.es.osaka-u.ac.jp


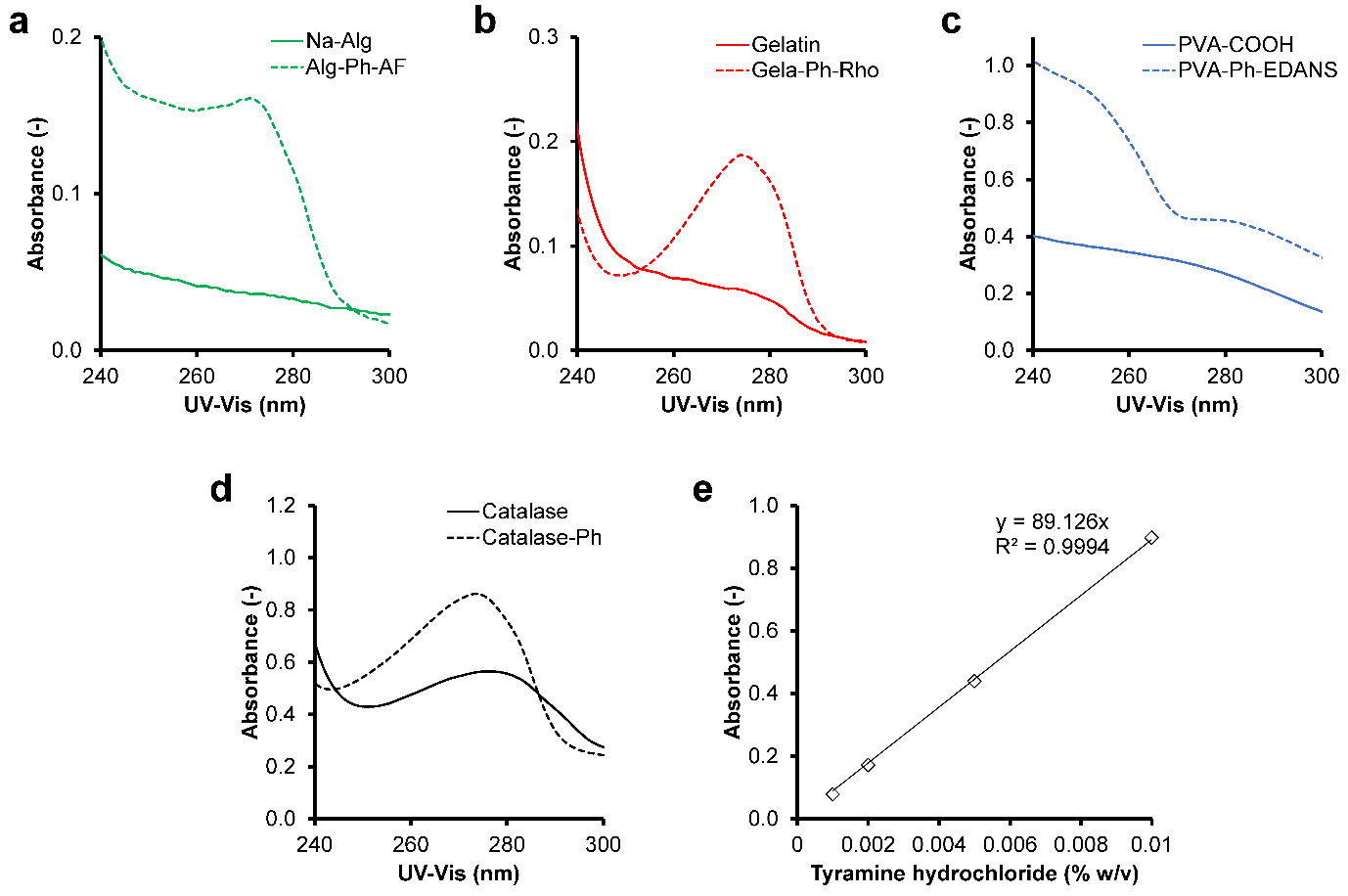


**Fig. S1.** UV-Vis spectra of polymer modified phenolic hydroxyl groups. (a) Alg-Ph-AF, (b) Gela-Ph-Rho, (c) PVA-Ph-EDANS, and (d) Catalase-Ph compared to the unmodified polymer. Note the peak at 275 nm in the Polymer-Ph. (e) Tyramine hydrochloride standard.


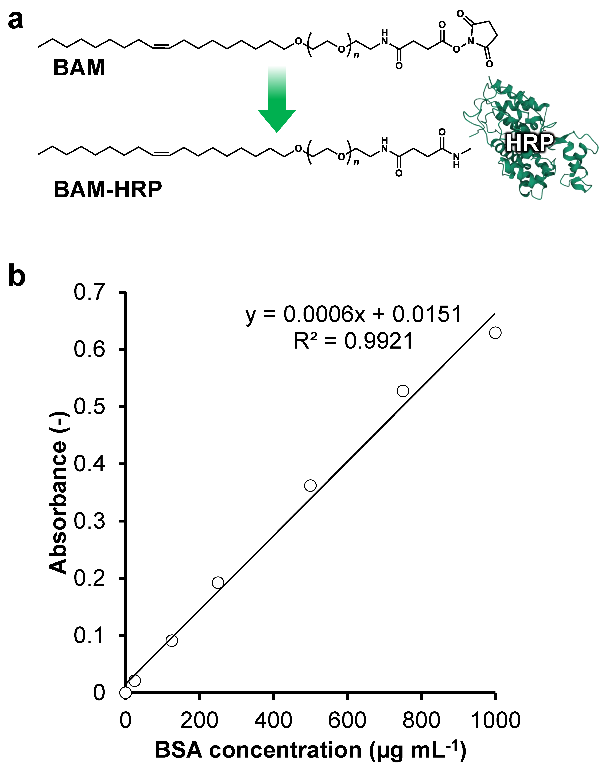


**Fig. S2.** (a) Conjugation of HRP to BAM. (b) Standard of bovine serum albumin (BSA) used to determine the concentration of BAM-HRP in Bradford Protein Assay.


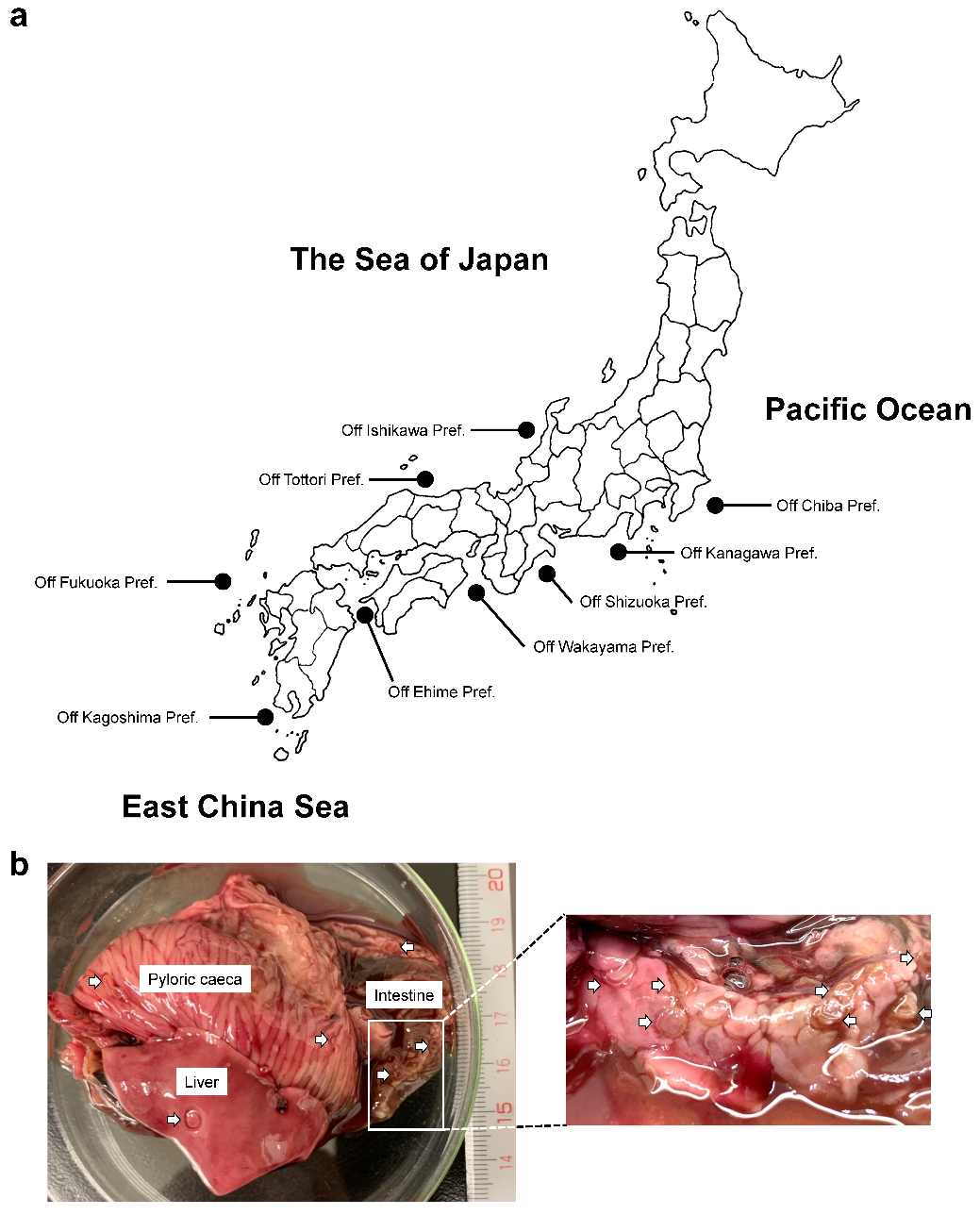


**Fig. S3.** *Anisakis simplex* isolation. (a) Geographical locations of host fishes used to isolate the *A. simplex*. (b) *A. simplex* (arrows) in the internal organ of fish.


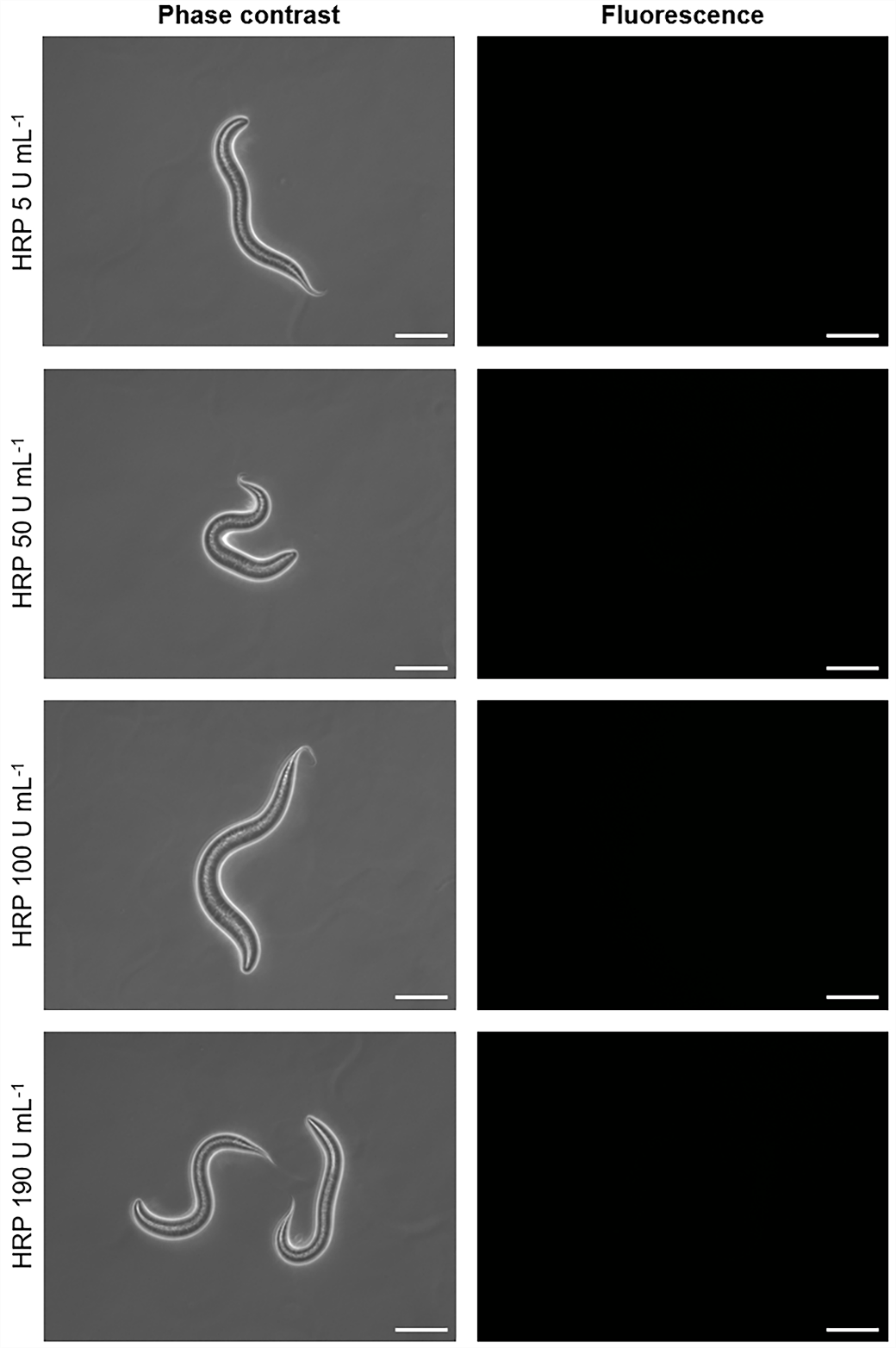


**Fig. S4**. Control test in which *C. elegans* was immersed in PBS solution containing 5, 50, 100, and 190 U mL^-1^ HRP without conjugation to BAM, followed by immersion in 1.0% w/v aminofluorescein-labeled Alginate-Ph (Alg-Ph-AF) + 0.1 mM H_2_O_2_. Scale bars: 100 µm.

**
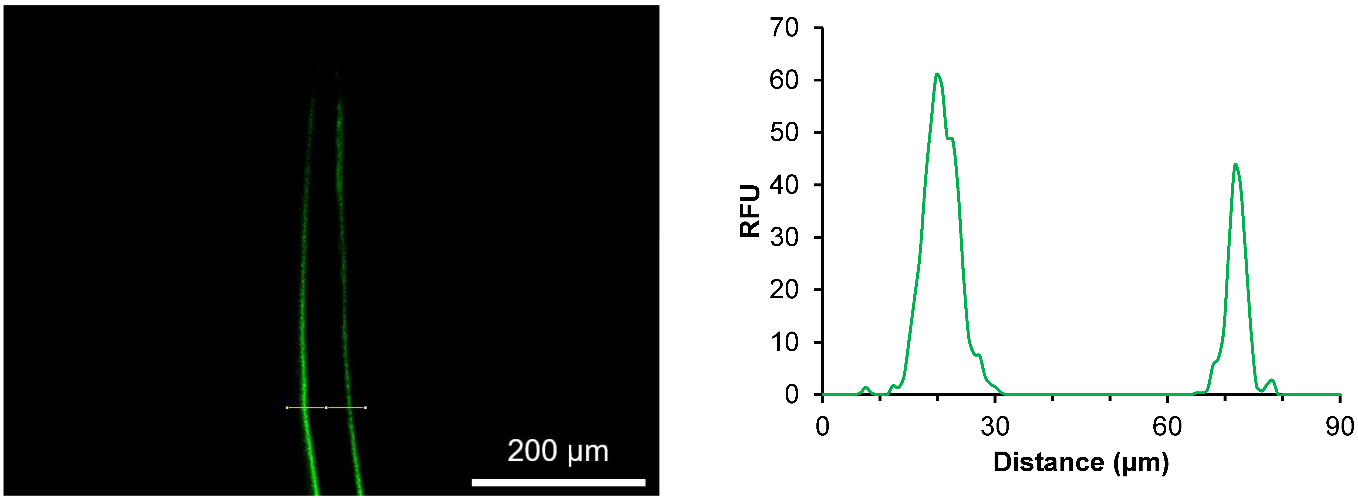
**

**Fig. S5.** Relative fluorescence intensity of the Alg-Ph-AF hydrogel sheath measured from the horizontal yellow line across the coated *C. elegans*.


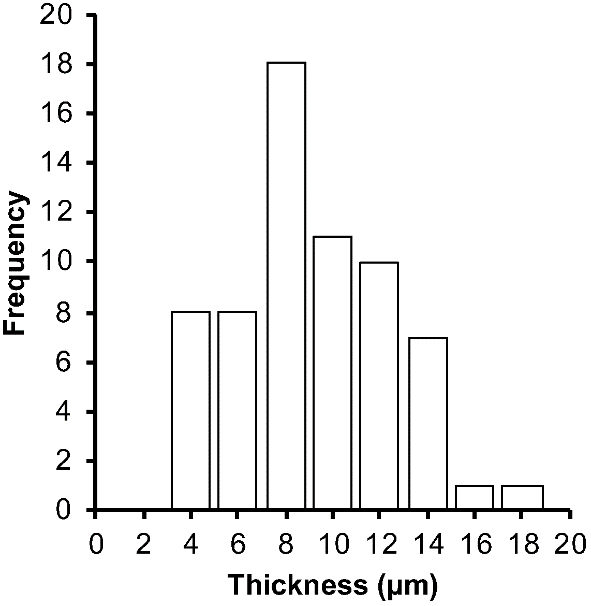


**Fig. S6**. Distribution of the hydrogel sheath thickness on the surface of *C. elegans.*


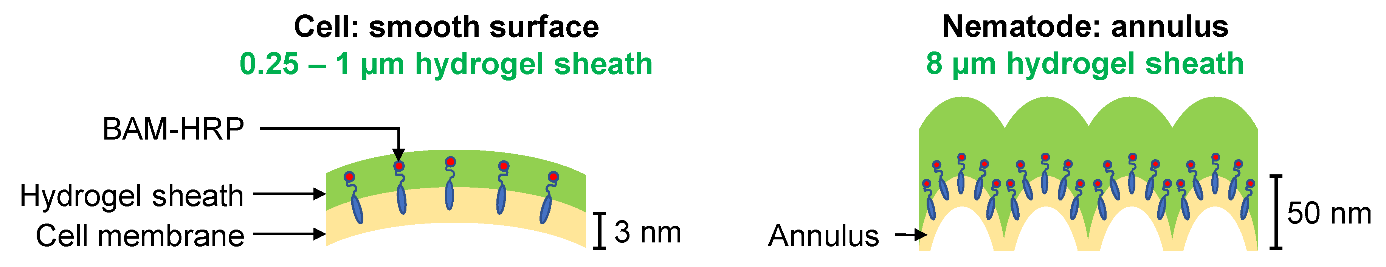


**Fig. S7**. Schematic illustration of the hydrogel sheath on the surface of cells and the nematode. Difference in the hydrogel sheath thickness might be caused by the difference in BAM-HRP density and annulus structure on the nematode.


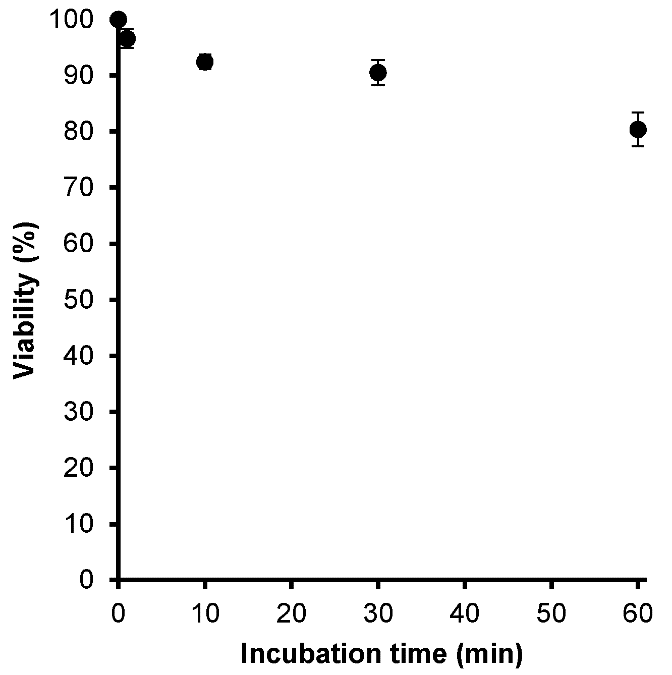


**Fig. S8**. Viability of *C. elegans* following immersion in 0.1 mM H_2_O_2_ solution for 60 min. Bars: S.E. (*n* = 5).


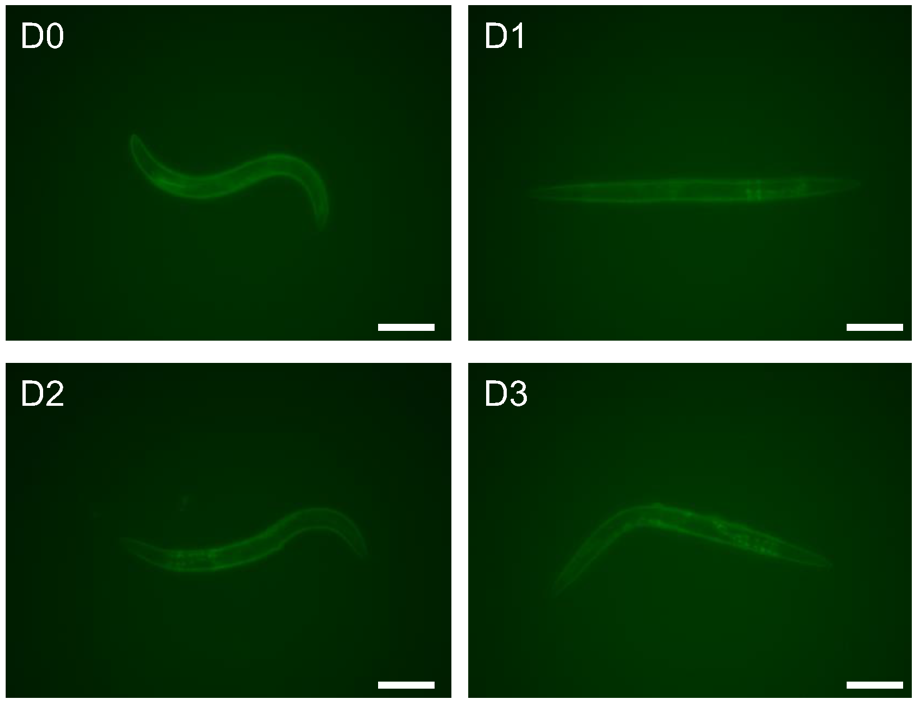


**Fig. S9**. Stability of Alg-Ph-AF hydrogel sheath on the surface of *C. elegans* cultured on nematode growth media (NGM) plate from day 0 (D0) through day 3 (D3). Scale bars: 100 µm.


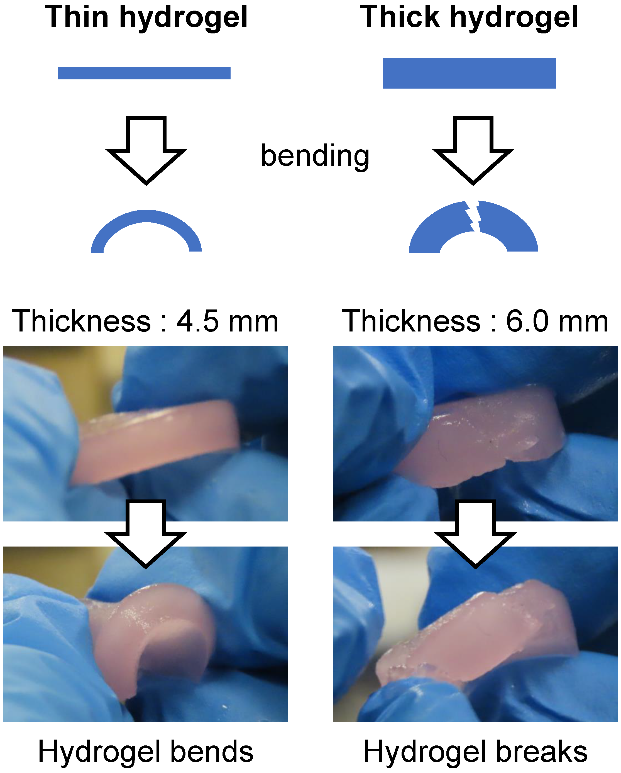


**Fig. S10.** The flexibility of thin and thick hydrogel. Notice the hydrogel with 6.0-mm thickness breaks upon bending.


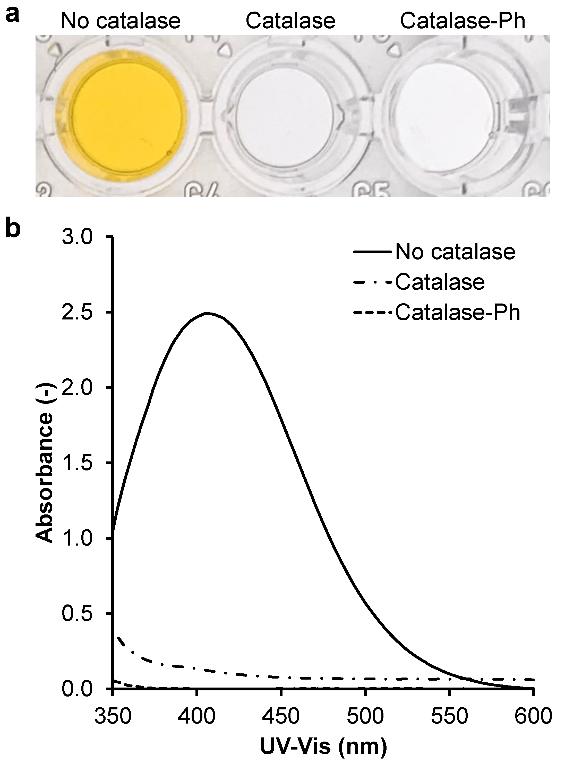


**Fig. S11.** Comparison of the activity of catalase and Catalase-Ph in degrading H_2_O_2_. (a) Colorimetric reaction of 10 mM H_2_O_2_ in dH_2_O after incubation with 1.0% w/v catalase or 1.0% w/v Catalase-Ph, and without catalase for 60 min in 37 °C with titanium (IV) sulfate (Ti(SO_4_)_2_) solution. Yellow color appeared in the sample without catalase due to the formation of titanic acid. (b) UV-Vis spectra (350–600 nm) of the sample mixed with Ti(SO_4_)_2_. Notice the low absorbance in 405 nm of H_2_O_2_ mixed with catalase and Catalase-Ph.


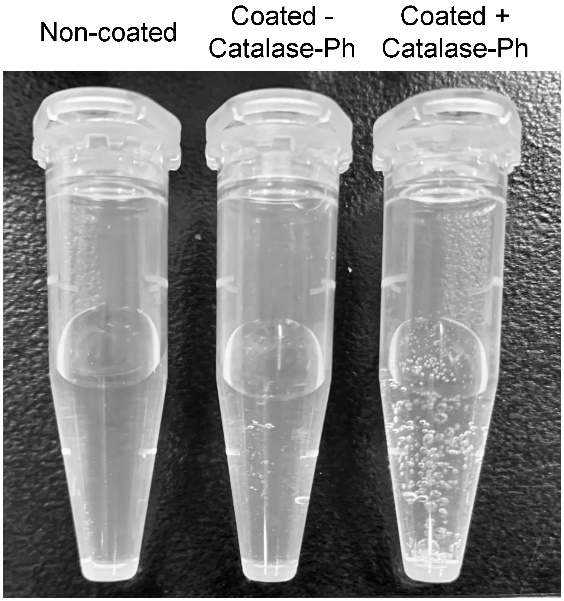


**Fig. S12.** Catalase-Ph activity in degrading the H_2_O_2_ to water and oxygen in the surrounding area of *C. elegans* coated with Alg-Ph-AF hydrogel loaded with 1.0% w/v Catalase-Ph. Notice the bubble formation only in solution containing *C. elegans* coated with gel loaded with Catalase-Ph.


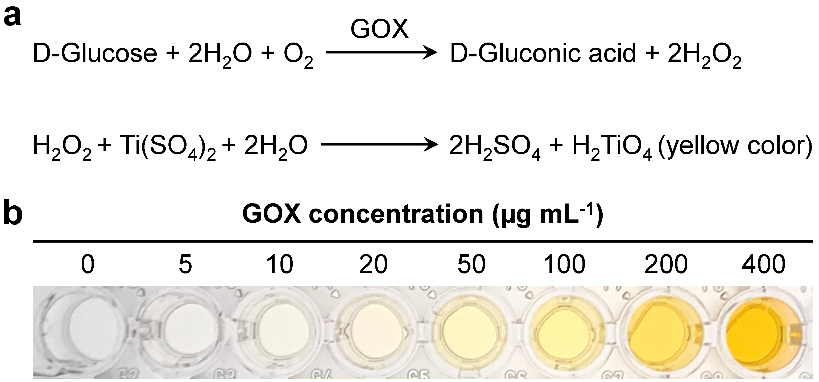


**Fig. S13.** H_2_O_2_ release by GOX. (a) Detection of H_2_O_2_ as a product of GOX activity using titanium sulfate. (b) Color changes of Ti(SO_4_)_2_ in the presence of H_2_O_2_ released by 0–400 µg mL^-1^ GOX mixed with 10 mg mL^-1^ D-glucose for 3 h. Yellow color indicates titanic acid formation after reaction with H_2_O_2_.


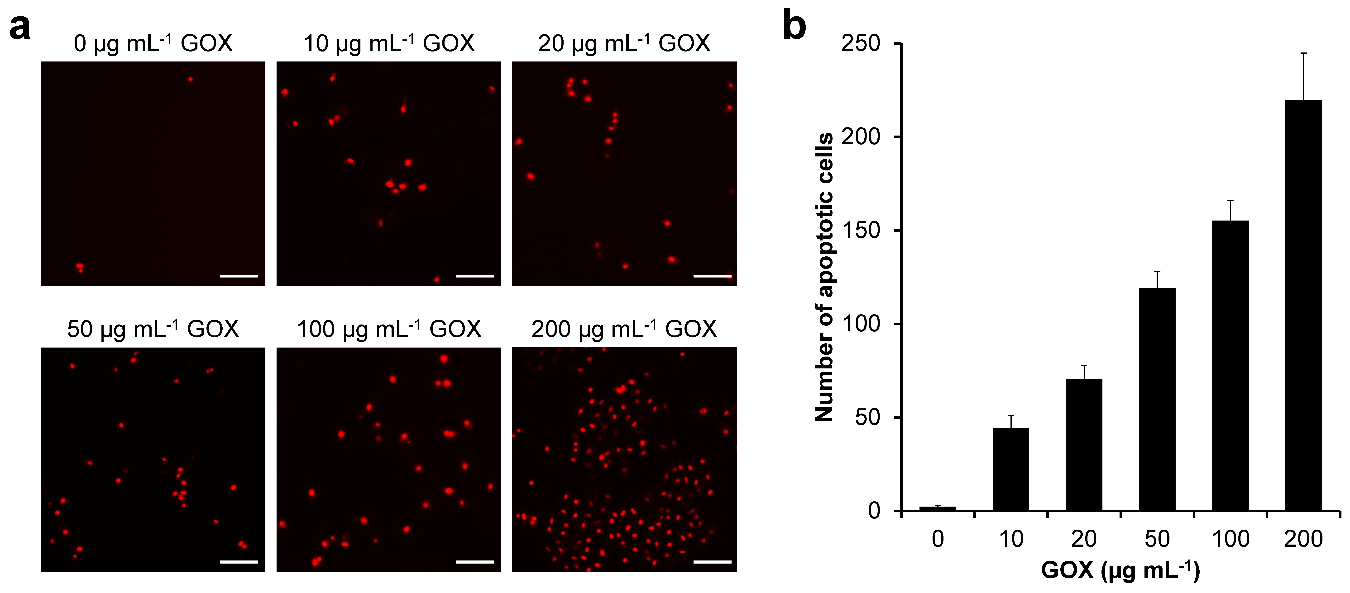


**Fig. S14.** Glucose oxidase induced concentration-dependent cell death of HeLa cells. (a) Representative fluorescence micrograph of HeLa cells exposed to 0 to 200 µg mL^-1^ glucose oxidase (GOX) stained with propidium iodide (PI). Scale bars: 100 µm. (b) Number of PI-stained cells. Bar: S.E. (*n* = 6).


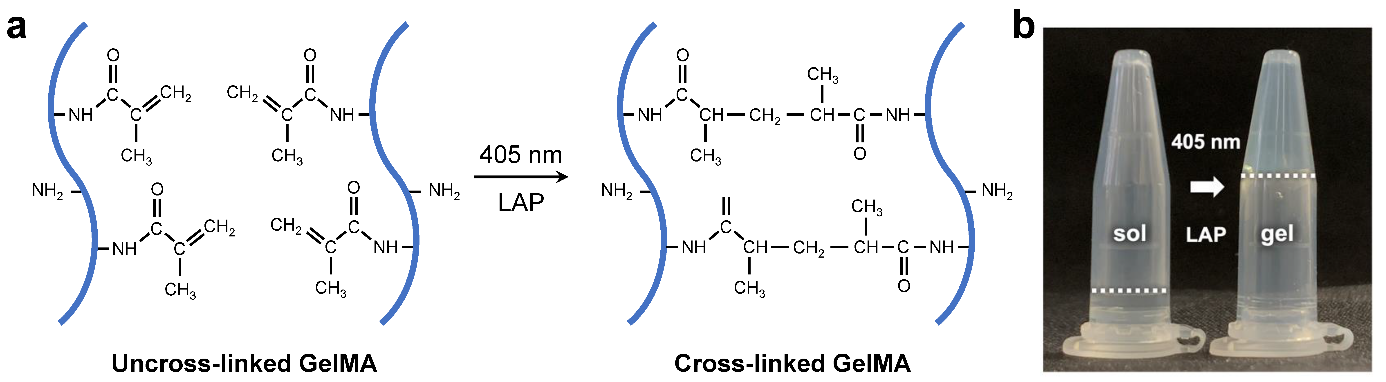


**Fig. S15.** (a) Gelatin methacrylate (GelMA) hydrogel fabricated from lithium phenyl-2,4,6-trimethylbenzoylphosphinate (LAP)-mediated photo-cross-linking following exposure to 405 nm blue light. (b) Hydrogel was fabricated from a solution containing 5.0% w/v GelMA and 0.5% w/v LAP followed by exposure with 405 nm blue light for 40 s.


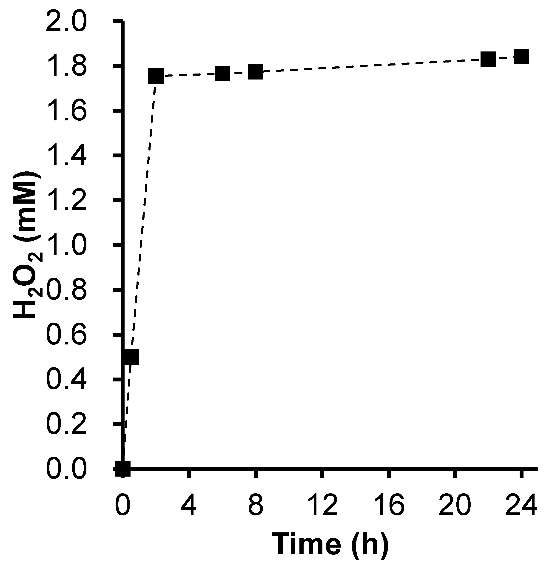


**Fig. S16.** H_2_O_2_ production by 100 µg mL^-1^ GOX in 1 mg mL^-1^ D-glucose. Bar: S.E. (*n* = 3).

**Mov. S1.** Comparison of locomotion of non-coated *C. elegans* and *C. elegans* coated with Alg-Ph-AF hydrogel sheath.

**Mov. S2.** Fluorescence observation of the movement of *C. elegans* coated with Alg-Ph-AF hydrogel sheath.
